# Influence of hearing loss on sibling relationships: Perspectives of the normal hearing sibling

**DOI:** 10.4102/sajcd.v70i1.939

**Published:** 2023-10-13

**Authors:** Suvishka Barath, Senamile N. Hlongwane, Marylene Madlala, Sinawo L. Mzanywa, Jessica Paken

**Affiliations:** 1 Department of Audiology, Faculty of Health Science, University of KwaZulu-Natal, Durban, South Africa

**Keywords:** siblings, hearing loss, aural rehabilitation, communication, family, relationship, South Africa

## Abstract

**Background:**

Sibling relationships, one of the most extended relationships in life, contribute to an individual’s social and emotional development. However, this relationship may be influenced if one sibling has a hearing loss.

**Objectives:**

This study explored the influence of a hearing loss on the sibling relationship by gaining the perspectives of the sibling with normal hearing.

**Method:**

Adopting a phenomenological research design, semi-structured online interviews were conducted using Zoom and WhatsApp with nine participants who have siblings with hearing loss.

**Results:**

Hearing loss may significantly impact the normal hearing sibling’s quality of life, affecting their psychological and social well-being, familial and peer relationships and overall experiences. Personal development, independence, maturity and closer sibling bonds were some of the positive influences reported by the participants. Attendance to aural rehabilitation therapy sessions, knowledge of hearing loss, personalities and household living arrangements positively influenced the relationship between siblings. The lack of attendance to aural rehabilitation sessions resulted in communication difficulties between siblings.

**Conclusion:**

There is a need to include siblings in aural rehabilitation and family engagements related to the child with hearing loss to provide a holistic and more effective rehabilitation and adjustment process.

**Contribution:**

This study aimed to improve family-centred intervention as it is focussing on the siblings’ perspectives of the hearing loss. Furthermore, previous studies have generally focussed on adults and not much research has been conducted surrounding the sibling relationship.

## Introduction

According to the World Health Organization ([WHO] 2021), approximately more than 1.5 billion people globally experience hearing difficulties, of which many children live in sub-Saharan Africa (Desalew et al., 2020). According to the American Academy of Pediatrics (2020), childhood hearing loss is one of the leading causes of the global burden of disease, with the highest rising prevalence in children younger than 1 year. Childhood hearing loss can negatively impact a child’s development. It can cause delays in speech, language, cognitive and literacy development (Pimperton & Kennedy, 2012). In addition, there is an impact on the family and larger community (Storbeck, 2012). According to Greeff and Van der Walt (2010), a diagnosis of hearing loss can come as a shock to the family and can have detrimental effects on marital relationships, family socialisation and everyday family routines. The presence of a childhood disability, such as hearing loss in a family unit, can alter the normal patterning of interactions between parents, parents and children, siblings and extended family members (Macker, 2015). According to Sahli and Belgin (2011), 80% of people in society have at least one sibling, suggesting that siblings play a crucial role in many individuals’ lives. Brotherhood and sisterhood can be the most extended relationships in life, resulting in the development of social and emotional well-being among siblings (Eichengreen & Zaidman-Zait, 2020). However, this relationship may be influenced if one sibling has a hearing loss.

The relationship between siblings contributes to young children’s language and cognitive development and their ability to understand other people’s emotions and perspectives while also contributing to their social development (Brody, 2004). Through these equal exchange interactions, younger children get opportunities to develop interpersonal and social skills, namely the ability to consider other people’s perspectives and negotiation (Tucker & Updegraff, 2009). A hypothesis on individual psychology puts siblings at the centre of an individual’s day-to-day life and character improvement (McHale et al., 2012). It was contended that social correlations and power dynamics in families, specifically sibling competition for family assets and resources, primarily affected character development. Siblings’ relationships are moulded by various factors, including childhood attributes, social standards and qualities (McHale et al., 2012). Less consideration has been provided for how siblings impact each other indirectly and their part as building blocks of the family structure (McHale et al., 2012). McHale et al. (2012) stated that the involvement of siblings in each other’s lives as companions, confidants and role models for social comparisons is very crucial and that their regular contact and close relationships during childhood and adolescence, especially without the immediate oversight of guardians or other adults provide abundant freedom to them and eventually shape their conduct and socioemotional advancements. McHale et al. (2012) further added that sibling relationships might make up for family conflicts, such as divorce or negativity. Close sibling relationships can protect youth from adjustment problems. Children’s time with their siblings is often more significant than the time spent with their parents (Ray, 2014). Therefore, a sibling relationship is one of the longest and most important relationships in an individual’s life, as it can be a source of learning. Furthermore, supportive sibling relationships can help guard against low self-esteem, depression and loneliness in the face of low parental and peer support (Milevsky & Levitt, 2005).

Studies focussing on the impact of childhood hearing loss on siblings and sibling relationships present conflicting findings. While some studies revealed a negative impact of having a sibling with a hearing loss (Raghuraman, 2008; Tattersall & Young, 2003), more recent studies show that these siblings can positively influence each other (Burke, 2010; Crowe, 2017; Emerson & Giallo, 2014). Additionally, a perusal of literature revealed a lack of studies focussing on the sibling of children with hearing loss in the South African context. Studies in this area of interest have been conducted in countries like the United States of America, the United Kingdom, Canada and Belgium. According to the United Nations (2020), these countries are classified as developed and high-income countries. Therefore, these studies may not be contextually relevant to the South African context, a developing and upper-middle-income country (United Nations, 2020).

This study may reveal novel findings in South Africa, a country where family dynamics may be significantly different due to single-parent and child-headed households. Furthermore, according to Stats SA (2016), about 26% of South African children live in child-headed households. Many children in South Africa are left without parents or primary caregivers due to the human immunodeficiency virus (HIV) and/or acquired immunodeficiency syndrome (AIDS) (Le Roux-Kemp, 2013). Currently, South Africa faces a quadruple burden of disease, which includes HIV and/or AIDS and tuberculosis (WHO, 2018). Therefore, according to Le Roux-Kemp (2013), it is common to have older siblings care for their younger siblings and sick parents. When there is a child with a hearing loss in the family, the older sibling may have an additional responsibility. It is, therefore, essential to describe the relationship between the siblings, considering this unique situation in South Africa. This difference in family dynamics may also be attributed to poverty. The significantly higher poverty than other developing countries with upper-middle-income status (Biyase & Zwane, 2018) can be partly attributed to the rise and persistence of unemployment (Cloete, 2015). Statistics South Africa (2020) reported that the unemployment rate has increased in the 4th quarter of 2020 (October–December) to 42.6% where almost one in every two people are unemployed in the country, often leading to internal labour migration, where people work far from home and thus cannot commute daily (Smit, 2001). As a result, many children are raised in single-parent households where parenting responsibilities are usually shared with another family member, such as the grandparent or an older sibling (Kautzky, 2009). Children in Southern African countries often have a significant contribution and role to play in the day-to-day running of the household, and children are often expected to play the role of caregivers in situations where a family member might be ill; this can include grandparents or siblings (Evans, 2010). Therefore, combined with the expectations to participate in a household’s productive and reproductive labour, caring for a sibling with hearing loss may be challenging.

Family members play a significant role in rehabilitating a person with hearing loss (Meyer et al., 2015). The child with hearing loss cannot be assisted without helping the family at the same time. It is crucial to consider the quality of life and the needs of the child and the family (Rhoades & Jill, 2010). Family-centred intervention accepts that both the person with hearing loss and those around them should be the focus of rehabilitation; therefore, this study will help professionals involve siblings in a meaningful way and to optimise opportunities to improve the quality of life of people with hearing loss as well as that of their families. This study will provide the audiologists working in South Africa with context-relevant data and alert them to the information that should be addressed with siblings during the family-centred intervention. It may reiterate the need to make the intervention more holistic so that the audiological issues and the psychological issues that may affect the child with the hearing loss are targeted. If the sibling relationship is tenuous, it may make the child with the hearing loss unhappy and isolate themselves from the family. Therefore, this served as an impetus for the current study exploring the normal hearing individual’s perspective of the influence of hearing loss on their sibling relationship, including their psychological and social well-being. This study will incorporate the relationships and interaction of the normal hearing siblings with their peers, parents and other family members.

**FIGURE 1:**
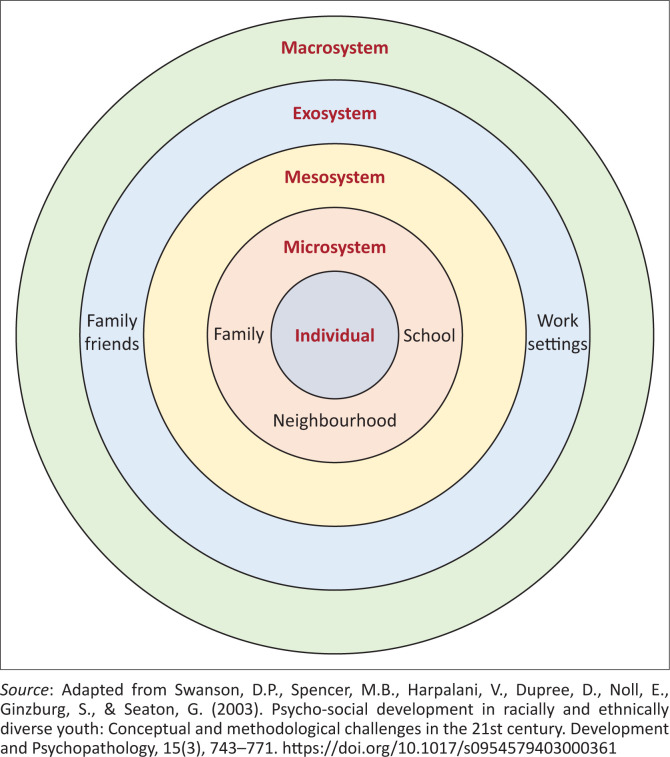
Bronfenbrenner’s ecological system theory.

The conceptual framework employed in this study is aligned with Bronfenbrenner’s ecological systems approach (Swanson et al., 2003). This theoretical framework draws attention to the larger environment where sibling interactions and relations occur and develop (Whiteman et al., 2011). According to this framework, individual development is influenced by four different systems, namely micro-, meso-, exo- and macrosystems, as reflected in Figure 1.

Microsystems can be defined as contexts in which a person has primary face-to-face contact with essential and impactful individuals, including family members and neighbours, in their everyday life (Hooper & Crusto, 2013). Mesosystems are continuous interactions between microsystems. Therefore, what happens in the family can influence peer and sibling relationships (Bishop, 2012). Exosystems refer to systems in which an individual is not directly engaged but influences the people with proximal relationships, indirectly influencing them (Bishop, 2012). Macrosystem refers to ‘the broadest and highest-level system, consisting of overarching societal, social, cultural, political, institutional, and procedural beliefs, values, and components’ (Hooper & Crusto, 2013, p. 2). Each system operates interdependently; hence, the researchers aim to describe how factors within these systems affect the sibling relationship. Therefore, the research question of this study is ‘How does hearing loss influence the sibling relationship?’

## Research methods and design

### Research aim and objectives

This study aimed to explore the influence of hearing loss on the sibling relationship. The following objectives were formulated to achieve the aim of the study:

- To explore the influence of an individual’s hearing loss on the normal hearing siblings’ psychological and social well-being.
- To describe the interactions between the individual who has a hearing loss and his or her normal hearing sibling.
- To describe the experiences of the normal hearing siblings due to having a sibling with hearing loss.
- To describe factors that influence the relationship between siblings, one of whom has a hearing loss.

### Study design

A qualitative research approach using a phenomenological design was used to conduct this study. This research approach was appropriate for this study as the researchers sought to explore the experiences and perspectives of individuals with siblings who have hearing loss. The type of phenomenological design that was identified for this study is transcendental phenomenology. Transcendental phenomenology is a philosophical approach to qualitative research methodology to understand human experience (Sheehan, 2014).

### Setting

The research study took place in different provinces in South Africa, namely KwaZulu-Natal, Eastern Cape and Gauteng.

### Study population and sampling strategy

The study included normal hearing siblings of individuals with hearing loss. Nine participants were interviewed. Participants were recruited through snowball sampling. ‘Snowball sampling, also known as chain referral sampling, is a non-probability method of survey sampling selection that is commonly used to locate rare or difficult to find population’ (Johnson, 2014, para. 1). The researchers requested the selected participants to provide referrals to recruit other eligible participants for the research study. Thereafter, purposive sampling, using the following selection criteria, was employed.

#### Inclusion criteria

- Individuals who were older than 12 years of age, as these participants were able to provide more in-depth responses to the questions.
- The sibling can be older or younger, as this will provide information on whether being older or younger than the individual with a hearing loss influences the sibling relationship.
- Participants who self-reported normal hearing, as this study specifically focuses on gathering information from the perspective of normal hearing siblings. Therefore, siblings who also present with a hearing loss were excluded.
- Both females and males were included as participants, as experiences may differ depending on their roles in the family.
- The sibling can present with a hearing loss of any degree, as the experiences and impact on the sibling relationship may be different depending on the degree of hearing loss.

#### Exclusion criteria

- The sibling should not present with other impairments, except for hearing loss. Presenting with additional impairments may compound the effect of hearing loss on the sibling relationship.

### Data collection

The researchers used a semi-structured interview schedule (Appendix, Table A-1), adapted from Eichengreen and Zaidman-Zait (2020), to conduct an online interview using Zoom and WhatsApp video calling. Samsung Galaxy A51 mobile phone, which has a 48-megapixel camera, was used to record the laptop’s screen during the WhatsApp video call. The interview schedule was also translated into isiZulu, as it is the most commonly spoken first language in South Africa (SAfacts, 2022).

### Data analysis

Deductive thematic analysis was used to analyse the data. The analysis was conducted using NVivo 12, a qualitative data analysis software (QDAS). The six-step analysis process developed by Braun and Clarke (2006) was followed to analyse the data in this study.

### Reliability and validity

Before the main study, the researchers conducted a pilot study with two individuals (aged 18–25 years). The results obtained from the pilot study indicated that the data collection tool had to be amended. Two researchers had to record the interview at the same time in the event of internet connectivity problems.

The chosen methodology allowed the researchers to collect data within the appropriate context regarding the cultural and linguistic variables (Leung, 2015). This was achieved using tools and documents, which were also translated into isiZulu. Tools or documents used during the data collection process were translated to isiZulu and then back-translated to English by two individuals (working independently) to ensure the accuracy of the translation.

Construct validity was ensured in the study through the pilot study, to allow for the researcher to be cognisant of any changes that needed to be made while conducting the interview. The tool was adapted from Eichengreen and Zaidman-Zait (2020), further adding to the construct validity.

During data analysis, the researchers engaged with each other to reduce bias; the transcriptions were swapped between the researchers, and a cross-check was conducted. The researchers also ensured reliability and validity through respondent validation. As the participants were either in employment or currently attending high school and tertiary institutions, their interview transcriptions were sent to each participant to allow the participant to comment on its accuracy (Noble & Smith, 2015). All participants confirmed the validity of the interview transcriptions.

### Ethical considerations

This research study abided by the ethical principles, outlined in Miller et al. (Eds. 2012). Prior to data collection, ethical clearance was obtained from the University of KwaZulu-Natal’s Human and Social Sciences Ethics Committee (HSSREC/00002835/2021). Privacy and confidentiality were maintained for all participants of this study. In addition, participants were assigned pseudonyms, and no personal names or information were used in the study. Written informed consent was obtained from all the participants. Parental consent was obtained for children under the age of 18 years. The child was also required to provide assent. Participants were informed that they could refuse or withdraw participation from the study at any given time without any consequences.

### Description of participants

Nine participants, comprising four females and five males between the ages of 14 and 31 years, contributed data to the study. A brief description of the participants with their assigned pseudonyms is as follows:

- Mbali is a 14-year-old, grade nine female from Durban, KwaZulu-Natal, with an older deaf sister. She communicates in sign language with her sister.
- Natasha is a 31-year-old female audiologist from Durban, KwaZulu-Natal, who has an older brother who is deaf. Her brother contracted congenital rubella and now has a profound hearing loss. He belongs to the deaf community and uses sign language to communicate.
- Mandla is a 19-year-old male university student whose younger sister is currently fitted with a unilateral cochlear implant. They use only the oral method of communication.
- Musa, a 19-year-old male residing in Soweto, has a younger sibling fitted with cochlear implants. He currently uses sign language to communicate with his sibling.
- Susi is a 15-year-old female who is currently in school. She has a 9-year-old sibling who is congenitally deaf and was fitted with a cochlear implant on one ear and wears a hearing aid on the other. They use total communication to communicate.
- Zandy is a 16-year-old female who is currently in high school. She has a 6-year-old sibling who is fitted with a unilateral cochlear implant. They are currently using total communication as a mode of communication.
- Andrew is a 23-year-old male university student who resides at his university residence and spends most of his time with his friends rather than his family. His brother is also 23 years old, and he is deaf. He uses sign language and written language to communicate, and he was fitted with bilateral behind-the-ear hearing aids when he was younger.
- Sipho is a 15-year-old male who is still in high school. His 6-year-old sister was born deaf and was fitted with a cochlear implant on her right ear only. She can talk and does not use sign language. The sibling with the hearing loss also has a twin brother who is normal hearing.
- Ntokozo is a 23-year-old musician. His 6-year-old brother acquired a hearing loss when he was 3 years old due to meningitis. He is profoundly deaf; he mumbles and produces single words and/or utterances to communicate. He was recently fitted with a unilateral cochlear implant.

## Results

**FIGURE 2:**
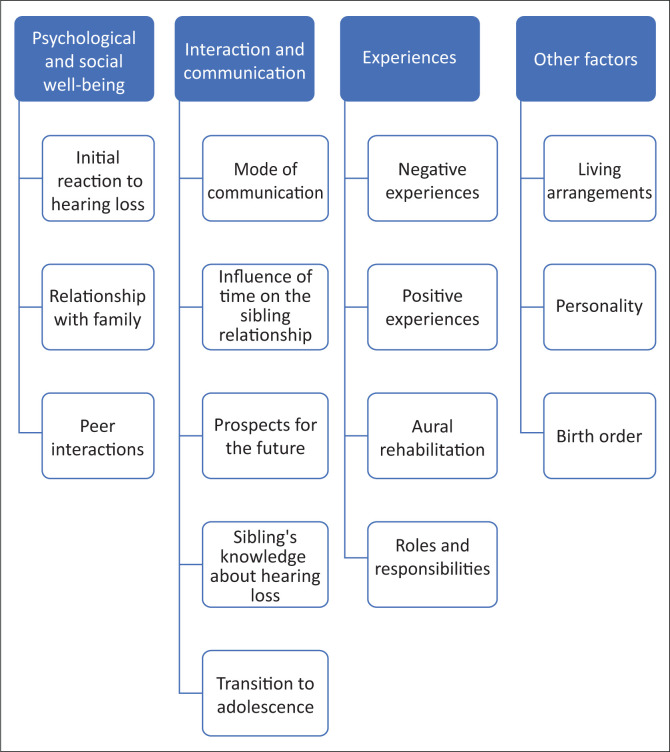
Hierarchy of themes and subthemes identified.

The results presented under each theme are English quotes from the participants. The quotes were provided verbatim in isiZulu; however, the translation is provided in English. The researchers identified four distinct themes from the research study. A hierarchy of the themes identified from the results is indicated in Figure 2.

### Theme 1: Psychological and social well-being

#### Initial reaction to hearing loss

In the current study, most participants reported their initial reaction to their sibling’s diagnosis being sadness, hopelessness and disappointment as their sibling does not meet their hopes and expectations. The participants experienced grief for the loss of what they hoped for and imagined their siblings would be.

Musa expressed a significant sense of loss:

‘I was heartbroken, yes, I thought that I would have a brother who will be like me. It would be possible for him to hear, but it was not like that.’

Participants also reported feeling surprised and hurt because their sibling was diagnosed with hearing loss at such a young age. The diagnosis of hearing loss brings about many emotions from the siblings. Some of the participant’s initial feelings may be associated with Kübler-Ross & Kessler (2009) five stages of grief: denial, anger, bargaining, depression and acceptance. The feelings of sadness, pain and hopelessness may be associated with the depression and the denial stage of grief. The first stage of grief, denial, is the initial feeling of disbelief and shock. Klotz (2018) reports that Kübler-Ross describes those experiencing this stage as being ‘paralyzed with shock or blanketed with numbness’. This is evident in Susi’s statement:

‘I don’t know how I really felt at that time.’

Siblings go through different stages of grief. They also undergo many psychological processes upon discovering their sibling’s hearing loss (Pillay & Moonsamy, 2018). Emotions are changing processes related to an individual’s everyday life that affect their quality of life (Pillay & Moonsamy, 2018). Hence, finding out that a sibling has a hearing loss can be quite devastating for the normal hearing sibling, as indicated by the participants.

#### Relationship with family

Most participants reported that their sibling’s hearing loss has not affected or changed their relationship with parents and extended family members in any way. Some participants felt like their parent’s attention was primarily focussed on the sibling with the hearing loss. However, that did not affect their relationship as they expressed that they understood that their sibling needed more attention due to the hearing loss. The research findings indicate that the sibling shows maturity and a sense of understanding towards their sibling with a hearing loss. Furthermore, Zandy expressed the following sentiments:

‘I think it was always just there and also by them (family) explaining to me and also my grandmother saying, “You the one who’s supposed to be there for her, she’s your sister, if not who’s going to be there for her and what not”. I feel I had to think that through, and you know get used to that.’

Hence the participant’s familial support greatly assisted in moulding her relationship with her sibling. However, Susi expressed:

‘I feel maybe if my mother had explained to me that she is deaf right now and she has to go to a totally different school, that was never explained to me at that time because a lot of attention was going towards her.’

From the above response, the effect of the family on the sibling relationship is evident as parents’ failure to apply equitable rules in the family towards their children may give rise to grievances raised by the normal hearing siblings about unfairness (Antonopoulou et al., 2012). Parents who cannot implement equitable rules in the family towards their children may provoke complaints, negative feelings and disruptive behaviour within the family. In our research findings, the family members who set rules and explained to a child about a sibling with a hearing loss resulted in a more cordial and trusting relationship as the normal hearing sibling understood their role and responsibility.

#### Peer interactions

In the current study, participants reported that they felt like their lives were different from their friends as they did not have a sibling with hearing loss and therefore did not have any insight on living with a person with hearing loss. Most participants reported that having a sibling with hearing loss did not change how they interacted with their peers and friends. They further reported that they did not perceive themselves to be any different from their peers due to having a sibling with hearing loss but reported that their friends do not always understand what it means to have a sibling with hearing loss.

Susi expressed:

‘Sometime when we are around friends, my friends don’t really understand what it’s like having a hearing-impaired sister. So, I have to explain to them this is how she does things, talk to her in this way or do this and that or if you do this, it makes her feel insecure.’

The above response shows that the participant is not embarrassed by her peers’ comments or questions regarding her sibling’s hearing loss. The participant elaborates on how she handles public encounters by briefly explaining hearing loss and communication strategies; hence, she advocates for her sibling.

Most participants reported that having a sibling with hearing loss did not affect their interactions with their friends in any way. They reported that their friends are often curious about their sibling with hearing loss and often show a willingness to learn more about their sibling’s disability; they even go as far as asking to be taught ways to communicate with this sibling.

Mbali expressed:

‘They’d be like, shocked and you know. And number two is most of the people just want to learn sign language.’

The normal hearing siblings’ peers may serve as role models or as focal points for self-reflection and self-assertion about coping with a sibling who has a hearing loss, thus positively influencing the sibling relationship (Eichengreen & Zaidman-Zait, 2020).

Bat-Chava and Martin (2002) state that (1) the differential amounts of attention received from family members (documented in our research findings), (2) the establishment of the hearing-impaired sibling as ‘different’ from other family members and (3) the failure to develop effective communication strategies by the normal hearing sibling in social settings may all result in a more distant relationship between these siblings. However, current research findings indicate that these factors result in a closer sibling bond, thus positively impacting the sibling relationship. It may be that those normal hearing siblings with higher rates of emotional, psychological and social well-being are more likely to communicate and adapt to their siblings and thus experience higher levels of sibling closeness (LeBouef & Dworkin, 2021).

### Theme 2: Interaction and communication

#### Mode of communication

Participants highlight the critical role of having a similar mode of communication for meaningful conversations. A similar communication mode between siblings creates more meaningful conversations between them. Andrew states that he and his family use sign language and written language (which is sometimes affected by the choice of language) and visual aids to communicate with his sibling:

‘Sometimes we talk to him using WhatsApp, but the problem is we can only chat using English because he can’t write in isiZulu. My family knows sign language; they are about five of them that can communicate with him fluently. Another way of communicating with him is by showing him something or writing something down on paper; in that way, he is able to understand and respond back.’

However, Andrew further mentions that when he wants to confide in his sibling, communication breakdown sometimes occurs, negatively impacting their relationship:

‘There will be a time where I want to communicate with him as someone who is also a male, but you find that you cannot just talk to him because you end up speaking to yourself, sometimes you try to explain things to him using sign language, but you see that he will be just agreeing so that he will not disappoint you.’

Natasha expressed that her sibling uses total communication, which helped their relationship, as he can relate to the hearing community and the deaf community. ‘… he uses total communication, so he’s able to associate with both worlds, you know, the deaf world and the hearing world. I think that really helped him’.

However, Natasha also commented that because her sibling uses sign language, he experiences difficulties understanding colloquial language, requiring her to serve as an interpreter for him in such situations.

‘If we were having big group conversations, you know, I used to interpret quite a lot or explain situations to him a lot. In the deaf culture, certain things they don’t really understand like slang, for example. Like Indian people use a lot of slang, so like Indian boys and things like that use a lot of slang, so sometimes he didn’t understand those things, because it wasn’t part of their vocabulary. So that was the thing that stood out because I kind of grew up in this whole environment I think I understood him the most.’

#### Influence of time on the sibling relationship

Participants indicated that they began to develop a more profound knowledge of their sibling’s hearing loss as time progressed, resulting in more communication and interaction. Hence, an increase in time drastically improved the sibling relationship as it resulted in most participants developing a stronger bond. Natasha states that she has become more accepting of her responsibilities towards her brother as she matured: ‘It is just something that they, you know, and you have to do, or you are not forced to do it, but you do it’.

It is, thus, evident that the participants feel like with growth came maturity and a newfound appreciation for their role in their sibling’s life.

#### Knowledge about sibling’s hearing loss

A lack of knowledge about their sibling’s hearing loss resulted in the participants being left out during crucial milestones in their siblings’ life, as expressed by Susi:

‘I feel maybe if my mother had explained to me that she is deaf right now and she has to go to a totally different school, that was never explained to me at that time because a lot of attention was going towards her. Yeah, I was never really part of that happening at that particular moment. I wasn’t a part of the school process, the cochlear and how the hearing aids are supposed to work.’

Ntokozo expressed that his mother has been instrumental in providing the necessary knowledge to him and has been the primary influence in him understanding his sibling better:

‘My mom told me everything I wanted to know. I think so because of her, I was able to understand my brother better.’

Natasha expressed that being knowledgeable about her sibling’s hearing loss was not of utmost importance to her as she was the younger sibling; hence, it did not affect her relationship with her sibling.

‘To me, it didn’t really matter, and quite honestly, the reasoning behind him getting there, or the degree of his hearing loss and things like that didn’t really matter, he was there, so there wasn’t anything we could really do, unless, and we had to adjust to him and he had to adjust to us in that way, but because I’m the younger sibling, it’s just something that was there already, there wasn’t anything I needed to know extra, you know.’

The younger normal hearing child is born into a typical routine, and there is no need to adjust from a previous, different lifestyle like older normal hearing siblings may have to. Therefore, this concurs with Natasha’s statement, suggesting that she joined her family’s natural flow of life. Having a sibling with hearing loss can be confusing and challenging for normal hearing siblings, especially if they have no prior experience dealing with any disability. It is evident that including the older siblings in the intervention of the sibling with the hearing loss leads to better relations.

The interactions between siblings play an important role in developing social skills and the cognitive and emotional development of siblings (Poole, 2009). Hearing loss heavily affects the communication and interaction between siblings. The effectiveness of the communication relies on the sibling’s ability to understand each other. According to Smith et al. (2013), a barrier in communication may negatively affect the sibling relationship, while using a similar communication mode leads to more enhanced exchanges with their sibling (Ray, 2014). Therefore, this encourages a closer relationship between the siblings. The findings from this study suggest that a difference in communication mode may cause a communication breakdown and lead to poor interaction between the siblings. The research findings also revealed that normal hearing individuals would often adapt and adjust to a different communication mode to communicate with their siblings with hearing loss more effectively.

#### Transition to adolescence

Individuals who transition to adolescence spend less time with their siblings and start to show a lack of respect as expressed by Mandla:

‘It has changed. At first, he respected me; now, he doesn’t respect me at all. He thinks he’s big. It doesn’t make me feel sad because it’s part of growing up; he has to grow up, and sometimes he has to be by himself. You know he has to learn a lot of things in life by himself without me being there.’

Susi reported a change in her siblings’ behaviour; however, this helped their relationship as they are now able to share a more intimate relationship:

‘So much has changed a lot. She has changed, she is very close to teenage-hood now, so we became closer. Because when she was younger, we were never that close. She was closer to her little sister. So now that she is growing, we are becoming closer.’

The above statement indicates that the age difference between her and her sibling has impacted their closeness and their relationship and that the transition to adolescence has fostered a more intimate and meaningful relationship. The interactions between siblings may change as the sibling relationship changes over time (Yeh & Lempers, 2004). The study findings revealed that sibling relationships become more meaningful with time, the interactions between siblings improved, and participants reported feeling much closer to siblings with hearing loss. This concurs with Raghuraman (2008), who reported that their feelings may change as siblings grow and have a more mature perspective. The interactions were also positively affected by the transition to adolescence. The findings revealed that while the transition to adolescence brought the siblings closer together, the opposite was true for one participant who reported that their interactions with their sibling were negatively affected. The participants in this study revealed that a lack of knowledge about their sibling’s hearing loss leads to a lack of understanding and thus negatively affects their interactions with their siblings. This finding concurs with Evans et al. (2001), who stated that the lack of knowledge in siblings of children with a disability might lead to poor interactions between siblings.

#### Prospects for the future

Participants expressed deep concern for their sibling’s future. They were concerned that their siblings would be discriminated against and lack confidence; hence, they would like their sibling to be independent. Participants indicated that they do not want their sibling to lose their sense of individuality and always be themselves regardless of the circumstances. In many participants’ responses, it can be seen that they wish for their sibling to be successful, happy, and have a career as seen in Susi’s statement:

‘I wish she becomes a great successful person, and she has things that belong to her because I mean the world is not going to be so friendly to her because she is deaf. So, she needs to learn how to do things for herself and not mind what people are saying about her and has confidence.’

These findings reveal that the participants are aware of the difficulties that their siblings may face in the future due to their hearing loss. The participants are worried about their sibling’s inclusiveness in employment, academic opportunities and inclusion in society. Furthermore, some participants have an ingrained sense of social justice and believe that society should not treat their siblings differently due to hearing loss.

### Theme 3: Experiences

#### Negative experiences

**Communication barriers:** In this study, participants described first-hand experiences of communication with their sibling with a hearing loss. Most participants understood the difficulties associated with hearing loss and had means of communicating with their siblings. However, the consensus from participants was that hearing loss often caused communication breakdowns. Communication between extended family members and the sibling with a hearing loss was also challenging, as reported by Musa, as he indicated that he often had to facilitate their conversations:

‘Yes, it did because they can’t communicate with him. Like they communicate with me. Yes, in order to get through to him, they have to talk to me or my mother first so that they can understand what he is saying.’

Some individuals may feel isolated or alone when people choose not to talk to them. This was supported by a statement made by Mbali suggesting that her sibling trusted and felt closer to her more than other family members.

‘You are the one that I trust more than the other people at home.’

Both communication partners can be affected significantly; it can also reduce the interaction between the two in an attempt to avoid conflict. In this study, however, participants made various attempts to maintain communication with their siblings. They learned sign language and had the means to communicate with each other. The difficulties associated with communication were noted and addressed timeously. These attempts to improve communication positively influence the sibling relationship.

**Social gatherings:** In this study, the effect that hearing loss has on attendance at social gatherings is evident as seen in Sipho’s statement:

‘Yes, we do consider her because maybe sometimes we’re going to Zoo Lake to go swimming, but then we think of her because of her cochlear implant as it might get wet or something.’

This suggests that having a sibling with a hearing loss meant that participants and their families had to consider the type of family activities and social gatherings they attended. Therefore, if one sibling has a hearing loss, the choice of activities they do together may be dependent on the affected sibling. Normal hearing siblings may not be able to participate in activities that they enjoy/prefer, which might lead to feelings of anger and jealousy as normal hearing siblings may begin to resent their sibling because they believe they receive preferential treatment (Yasgur, 2017), negatively affecting the sibling relationship as well.

#### Positive experiences

Some participants expressed that having a sibling with a hearing loss has helped them shape their character and be open to new experiences. Participants used various words to describe how having a sibling with a hearing loss has positively exposed them to a new world and personal development.

Mandla expressed:

‘I would say that it has made me a patient person or an understanding person, like now when I talk to her I can’t just say something once and expect her to understand, so I must keep saying something; it takes her a while to understand what someone is saying, so I have to repeat myself a lot.’

Although childhood hearing loss may be considered a challenge in the family, it also presents positive outcomes. In this study, participants were able to identify the positive influence their sibling’s hearing loss had on them. These include self-development, maturity, responsibility and competency. In addition to influencing their character and perspective on life, most siblings stated that they learned a new language/skill (i.e., sign language) because of having a sibling with a hearing loss.

#### Aural rehabilitation

When siblings of children with hearing loss are included in intervention and are provided with additional support such as sibling support groups, it increases their quality of life. Some participants mentioned that their siblings attended aural rehabilitation, and they were also involved in their therapy sessions. Natasha expressed that she was fully involved and active in aural rehabilitation therapy:

‘Yes, it was family centred intervention also. So there were sessions that not only my family, like my parents and myself, but it was his communication partners that were also involved like my cousins and my aunts, and everybody got involved in it, so it was quite nice in that sense.’

Ntokozo mentioned that he does not usually physically attend the aural rehabilitation sessions:

‘On those classes, to be honest, I’m 5% involved because when they do them, I’m usually not there so, unless if they want me to compile a video maybe of me teaching him maybe colours or telling him that this is a pen or something like that.’

The above statements indicate that a family-centred approach to intervention was followed for some participants and their families. Furthermore, some participants were well informed about hearing loss and its effect on their siblings because they attended aural rehabilitation. Susi mentioned that:

‘My mom started a society where we take in deaf kids like we take in the kids. We have activities because what I can tell you is that deaf people love being around other deaf people. Because that’s something they’re not used to. Yeah. That sense of belonging. Yes. And we have sign language classes. We’re trying to get sponsors.

We’re trying to get money to build a home where deaf people can stay.’

Natasha chose a career in Audiology because she was involved in her sibling’s aural rehabilitation therapy classes and developed an interest in Audiology at a young age:

‘Well, I mean, I’m an audiologist. I don’t think I would have thought about it if I didn’t have a hearing-impaired sibling. We’ve had a lot of opportunities I’ve had a lot of because he was very involved in a lot of things while growing up. I got a lot out of it. I also got a lot to understand the deaf culture, which helps me in many ways. Not only at work but just at home and in social settings.’

Essentially, this suggests that attending aural rehabilitation classes was considered beneficial and positively influenced these participants and their families. This is because in attempting to become closer and to alleviate some of the challenges experienced by their siblings, the participants ensured that they educated themselves and became involved in their sibling’s rehabilitation process. They were able to understand the different aspects of hearing loss better, and most importantly, they found a way to make a difference in their siblings’ lives and even their communities.

Both Andrew and Musa stated that their siblings attended aural rehabilitation. However, they were not involved for various reasons: the siblings attended boarding school and had their sessions there; hence, they could not attend and be involved. Additionally, some participants indicated that either their siblings did not attend therapy or did not know what aural rehabilitation therapy was.

In this study, three of the nine participants indicated that they attended aural rehabilitation with their siblings. In Andrew and Musa’s case, the accessibility of these services was an issue. Despite being probed, specific reasons for the lack of knowledge and non-involvement of other participants and their siblings were not disclosed.

The opposite was true in this study because participants who did not have enough information about their sibling’s hearing loss did not develop any assumptions. Instead, they believed that nothing was wrong with their sibling. However, this suggests that the parents of children with impairments must keep siblings updated and involved in their sibling’s management/rehabilitation. Therefore, the inclusion of siblings is encouraged for the effective intervention of children with hearing loss, especially when family-centred intervention is considered.

#### Roles and responsibilities

Participants indicated that they play vital roles in their hearing-impaired siblings’ lives. They are often asked to help out with the sibling who has a hearing loss or take on extra roles within the household. The findings of this study support this premise as participants reported that they were aware that their siblings received more parental attention, but they believe it to be justified. Participants who were both younger and older than their sibling with hearing loss reported having the responsibility of constantly looking after their siblings, especially when their parents were not around, as they took on the role of a guardian. Although participants understood their parents’ responsibilities, none of them reported feeling the need to be near-perfect or being responsible for compensating for their siblings.

Mbali and Sipho indicated that their sibling’s hearing loss does not influence their roles and responsibilities as they are only responsible for household chores and not their siblings:

‘Well, mostly normal stuff as a teenager as you know, cleaning all those stuff, house chores.’

Findings from this study suggest that roles and responsibilities of the normal hearing sibling vary within households. Hence, an overall increase in familial roles of individuals who are older than their sibling with hearing loss cannot be generalised.

**Daily functioning:** Participants indicated no drastic change in their daily functioning as their sibling’s hearing loss occasionally affects their lives.

Susi expressed:

‘It didn’t really affect my everyday life besides the fact that I must remind her to wear cochlear implants, and you need to explain things to her; she doesn’t understand.’

The participants believe that having a sibling with a hearing loss only had minor effects on their daily functioning. Although they had to make adjustments to accommodate their siblings, they did not indicate any major disadvantages of the hearing loss. Musa, however, indicated that there is a psychological effect on her daily functioning as she often worries about her sibling with a hearing loss being in situations that could cause him harm:

‘At some point like especially when I’m on the road. I get to feel like, he doesn’t get to hear the cars and I wonder how he is going to cope with hearing the cars hooting and when people ask him to move out the way, so I don’t know that’s how I think.’

Growing up with someone who has a disability of any sort can impact a person’s traits and characteristics. This influence can be both positive and negative and can push individuals to be better or cause difficulties. Participants in this study did not view their siblings as a burden. Andrew reported that his sibling’s hearing loss often affects his daily functioning; however, he did not express any frustration or anger regarding that. Alternatively, other participants reported that they made minor adjustments that did not affect their daily functioning significantly.

The findings from the study support a statement by WHO (2021), which stated that hearing loss in a family could disrupt many aspects of everyday life. Ray (2014) further stated that hearing loss could affect the hearing siblings’ quality of life and negatively influence the sibling relationship. The participant’s knowledge about their siblings’ hearing loss and communication mode played a vital role in facilitating effective communication between them and maintaining their relationships. Our research findings are congruent with Eichengreen and Zaidman-Zait (2020), who stated that families who use sign language to communicate with their children with hearing loss showed positive sibling relationships because normal hearing siblings contributed meaningfully to their sibling’s cognitive and socioemotional development. Persistent communication difficulties between siblings were attributed to the non-attendance to aural rehabilitation therapy classes and because normal hearing siblings did not receive informational counselling and strategies to improve the communication with their siblings with hearing loss.

In an attempt to avoid experiences that are one-sided and only guided by the sibling who has a hearing loss, parents should also be encouraged to attend and participate in activities that interest the normal hearing sibling and not allow the child’s needs with the disability to overshadow those of the normal hearing sibling (McHale et al., 2014). Siblings have various perspectives on their connection with each other. Older siblings are more likely to initiate interactions and educate younger siblings, and younger siblings are more likely to be taught and nurtured (Ray, 2014). However, in this study, it is evident that participants could learn something from their interactions with their siblings with hearing impairment, no matter the birth order. These interactions/experiences enriched the participants’ lives, and they appear to be more empathetic, mature and compassionate as a result (Caplan, 2011).

The inclusion in aural rehabilitation classes may be a way to create a healthier bond between individuals with hearing loss, their normal hearing siblings and their parents. According to Ray (2014), it is hoped that educators, audiologists and professionals will begin to think about the normal hearing siblings’ experiences and reflect more on their teaching practices, the assistance they provide to families, and how this could be improved when identifying and meeting families’ individual needs. Parents are also encouraged to spend one-on-one time with their normal hearing child regularly (McHale et al., 2014), as this will give siblings who feel left out the assurance that their parents truly love and care about them as individuals, and it will also lay the groundwork for healthy communication between parents and their normal hearing children.

### Theme 4: Other factors

#### Living arrangements

In this study, there were varying living arrangements. Participants did not attend the same school as their siblings. In most cases, the siblings with the hearing loss attended a boarding school for the deaf and only returned home during the weekends; hence, the siblings spent less time together. Participants who did not live in the same household with their siblings suggested that this influenced their relationship, and they identified a significant difference when they started spending more time together, as reported by Ntokozo:

‘We were not that close when this whole thing started happening. Another thing is, I wasn’t at home, like, I went to university, you know, stuff like that. So, obviously, there was no close relationship. So that grew along the way while we spent time together, you know, so it grew, and we became close.’

Participants believed that spending less time together harmed their relationship and influenced how close they became; alternatively, spending more time improved their relationship. This is evident in Ntokozo’s statement, where he expresses that he started being close to his sibling after they started spending more time together.

#### Personality

Participants described their siblings’ personalities or characters differently, and some described them in contrast to their personalities. When asked to describe his sibling’s personality, Ntokozo stated that:

‘He has a wild personality, fun, talkative, and he is an imaginator. He is that person that is always full of energy. I can’t even describe the energy, but it’s always on point.’

He further expressed that:

‘Another reason why I feel him and I are getting along very well is because when he passes that energy to me, I’m able to see that type of energy and reciprocate it.’

This suggests that having similar personalities may positively influence a relationship because it becomes easier for people to relate to each other; they start spending more time together, thus creating a closer bond.

Susi mentioned that her sister is an introvert, and she does not like going out in public:

‘She’s somebody who doesn’t like being around a lot of people. She gets tired of being in public for too long. So, I’m the one who’s supposed to be with her maybe when we are supposed to go to a wedding or some gathering in the family, and she would say, I don’t feel like going there, and I’m the one who’s supposed to stay behind and be with her.’

In this case, the sibling’s personality/character meant that she would depend on her sister when dealing with or avoiding social environments in certain situations. The participant’s understanding of her sibling’s character and what she considers uncomfortable further emphasises that having a sibling as a source of support can decrease low self-esteem, depression and loneliness (Crowe et al., 2015) and improve the overall relationship.

#### Birth order

Siblings have varied experiences, depending on whether they are the older or younger sibling. Susi described her role caring for her sibling with hearing loss:

‘I think it was always just there and also by them explaining to me and also my grandmother “You the one who’s supposed to be there for you, she’s your sister, if not who’s going to be there for her and what not”. I feel I had to think that through, and you know get used to that.’

Typically, an older sibling dominates, and the relationship is asymmetrical during childhood, especially when the younger sibling has a hearing loss. In this study, most participants are older than their siblings, which agrees with the statement as participants are dominant in their sibling relationships as they also play a vital role in caring for their young siblings.

The time siblings spend with one another might be a good measure of the quality of their relationship. It was discovered that even when one sibling has a disability, there is a significant level of interaction between siblings (Stoneman, 2001). According to Morry (2005), trait similarity predicts self-esteem and provides consistency in cognitions related to long-term compatibility. Higher levels of feeling understood and validated by the sibling, higher self-esteem and lesser loneliness are all linked to perceptions of trait similarity (Bell, 1993). It is possible that siblings having similar personalities lead to benefits from interacting, such as fewer conflicts and more pleasant encounters (Fehr, 1996). Younger siblings usually seek help and protection from an older sibling because they are more likely to provide encouragement, kindness, proximity and teamwork than a younger sibling (Buhrmester & Furman, 1990). Parents assign social roles to their children, such as assisting younger siblings in putting on their own clothes. For many younger siblings, caring for an older sibling with a disability is a common occurrence.

### Alignment of themes with Bronfenbrenner’s ecological systems theory

In this current study, each system and the themes identified in the various systems have a mutual relationship. Each theme reflects different systems and reveals how they are connected, how they interact with each other and how this influences the sibling relationship. The participants’ initial reaction to their sibling’s diagnosis included feelings of sadness, a sense of hopelessness and disappointment, thus affecting their psychological state. The above themes reflect the microsystem and the mesosystem. The themes of interaction and communication can also be found within the microsystem and the mesosystem.

**FIGURE 3:**
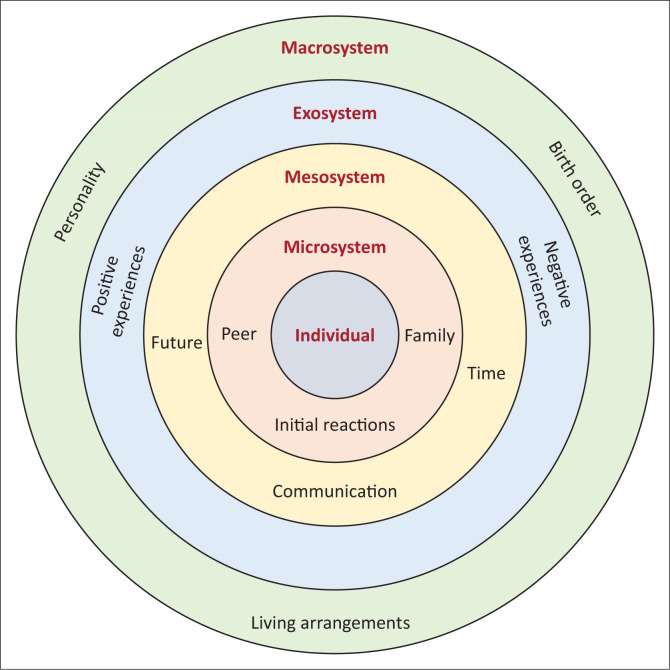
Alignment of themes to Bronfenbrenner’s ecological systems theory.

The sibling relationship may also be affected by informal and formal social structures outside the individuals and do not directly influence them. The participants in this study indicated some of the challenges regarding having a sibling with hearing loss, which included playing the interpreter’s role during social gatherings. According to Ray (2014), normal hearing siblings may feel frustrated with being the interpreter for their sibling’s interactions. This reveals that factors within the exosystem may also affect the sibling’s psychological state within the microsystem and the mesosystem. Therefore, the various systems can also affect each other. The participants in this study indicated that factors such as household living arrangements, personality and the birth order of the siblings affected their relationship. Participants in this study further revealed that different personalities and living arrangements affected the closeness in the sibling relationship. Therefore, the factors within the macrosystem can also affect the mesosystem and macrosystem as they affect the interactions between the siblings. The alignment of themes to Bronfenbrenner’s ecological systems is indicated in Figure 3.

According to Bronfenbrenner’s ecological systems theory, the sibling relationship is influenced by various contexts and the interaction of the factors within various contexts. Each system does not exist independently but is influenced by various systems.

## Conclusion

The experiences described by participants in this study differed as both positive and negative influences were observed. The effect of communication difficulties was identified throughout the interactions with participants. A common suggestion to alleviate these challenges was to include normal hearing siblings in aural rehabilitation services to develop skills that would enhance their interactions with their siblings with hearing loss. It is hoped that the research findings from this study shed light on improving the aural rehabilitation practices of audiologists and reflect the unique relationship that siblings share if one has a hearing loss.

## Appendix

**TABLE 1-A1:** The semi-structured interview schedule, adapted from Eichengreen and Zaidman-Zait (2020), to conduct an online interview using Zoom and WhatsApp video calling.

Number	Question
**Audiological history**	
1.	Currently, how old is your sibling?
2.	Was your sibling born with a hearing loss?
**Psychological and social wellbeing**	
3.	Describe your sibling’s personality or character.
4.	Discuss how you found out that your sibling has a hearing loss.
5.	Describe how you felt when you first noticed or were told that your sibling has a hearing loss.
6.	How has your sibling’s hearing loss influenced you as a person?
7.	How did your sibling’s hearing loss impact your relationship with your parents and other family members?
8.	How did your sibling’s hearing loss influence relationships with extended family members/community members/peers?
9.	Who do you go to for support when you are sad, upset, worried?
10.	What are your responsibilities in the house?
11.	How did your sibling’s hearing loss influence your friendships in the community/school if you attended the same school?
12.	How did your sibling’s hearing loss influence attendance to extra-mural activities-if applicable?
13.	How did your sibling’s hearing loss influence social gatherings, e.g. birthday parties, weddings, etc?
14.	How did your sibling’s hearing loss influence church/mosque/temple, etc attendance?
15.	Describe how friends/family may have influenced how you relate to your sibling.
**Interactions/Communications**	
16.	What would you like to have known about your sibling’s hearing loss when you were younger (to help understand your brother/sister)?
17.	Describe how you communicate with your sibling.
18.	How does the hearing loss influence your relationship with your sibling?
19.	How do you think your relationship with your sibling would be like without your sibling’s hearing loss?
20.	How has your relationship with your brother/sister changed over the years?
21.	How is your relationship with your normal hearing siblings different from your relationship with your sibling with hearing loss?
**Experiences**	
22.	Describe how your sibling’s diagnosis affected your daily functioning.
23.	Does your sibling attend aural rehabilitation therapy?
24.	How has your role changed within the family after your sibling was diagnosed with a hearing loss?
25.	How is your life different from your friends’ lives?
26.	Describe what you like/enjoy the most about having a sibling with a hearing loss.
27.	Describe what you like the least about having a sibling with a hearing loss.
**Other factors**	
28.	How would you describe your relationship with your sibling?
**Possible probing questions**	
2.	(If no). How old were they when they were diagnosed with the hearing loss?
4.	How is the way you feel about your sibling’s hearing loss now different from the way you felt about it then? (Probing for support question too).
6. a)	Compare your parent’s relationship with your sibling and you.
b)	Do you feel there are differences? If so, what are these differences?
7.a)	What activities do you like doing as a family?
b)	Whom do you have the closest relationship with?
9.a)	Would you like to have more support?
b)	What kind of support would you like?
10.	What happens if you don’t want to take on those responsibilities?
18. a)	What worries you about your brother/sister?
b)	What are your wishes for your brother/sister?
c)	What have you learned from your brother/sister?
23.	How involved are you?
25.	Do you ever feel left out?
28.	Why would you describe it like that? Or what has influenced your relationship to be that way?

*Source:* Eichengreen, A., & Zaidman-Zait, A. (2020). Relationships among deaf/hard-of-hearing siblings: Developing a sense of self. *Journal of Deaf Studies and Deaf Education, 25*(1), 43–54. https://doi.org/10.1093/deafed/enz038
